# In vivo acute toxicity of detoxified Fuzi (lateral root of Aconitum carmichaeli) after a traditional detoxification process

**DOI:** 10.17179/excli2018-1607

**Published:** 2018-08-31

**Authors:** Wan Sun, Bo Yan, Rongrong Wang, Fucun Liu, Zhengyan Hu, Li Zhou, Li Yan, Kang Zhou, Jiawei Huang, Peijian Tong, Letian Shan, Thomas Efferth

**Affiliations:** 1 The First Affiliated Hospital, Zhejiang Chinese Medical University, Hangzhou, China; 2 College of Pharmaceutical Science, Zhejiang Chinese Medical University, Hangzhou, China; 3 Changzheng Hospital, Second Military Medical University, Shanghai, China; 4 Zhejiang Provincial Center for Disease Control and Prevention, Hangzhou, China; 5 Department of Pharmaceutical Biology, Institute of Pharmacy and Biochemistry, Johannes Gutenberg University, Mainz, Germany

**Keywords:** acute toxicity, Aconitum carmichaeli, aconitine, zebrafish, UPLC-MS

## Abstract

Many herbs of traditional Chinese medicine (TCM) possess not only therapeutic efficacy, but also toxicity towards normal tissues. The herbal toxicities occasionally cause serious adverse events or even fatal poisoning due to the erroneous use of TCM herbs. *Fuzi* (lateral root of *Aconitum carmichaeli*) is such an herb with its toxic ingredient, aconites. Aconitine, mesaconitine, and hypaconitine are the main toxic components of *Fuzi*, which are hydrolyzed into non-toxic derivatives by water decoction. Therefore, long-time decoction was commonly applied as a traditional way to detoxify *Fuzi* before use. Nevertheless, recent clinical trials presorted on adverse events induced by long-time decocted *Fuzi*, putting some doubt on the safety of *Fuzi* after the traditional detoxification procedure. To thoroughly determine whether or not long-time decocted *Fuzi* was safe, we conducted *in vivo* acute toxicity assays using both rodent and zebrafish models and performed chemoprofile analyses using HPLC and UPLC-MS. The HPLC analysis showed that toxic aconitine components were hydrolyzed into benzoyl derivatives with increasing time of decoction. These aconitines were undetected by HPLC in *Fuzi* after 2 h-decoction (FZ-120), indicating seemingly non-toxicity of FZ-120. Unlike the non-decocted *Fuzi* (FZ-0) and 60 min-decocted *Fuzi* (FZ-60) with lethal toxicity, FZ-120 at 130 g/kg did not cause any deaths or side effects in mice regarding body weight and biochemical parameters. This seems to confirm safety of *Fuzi* after long-time decoction. However, histopathological observations revealed an abnormal liver phenotype and a significant decrease of the liver index following FZ-120 treatment, indicating a potential hepatoxicity of FZ-120. By using a zebrafish model, we observed that FZ-120 at a dose range from 288 to 896 μg/ml caused considerable adverse events including arrhythmia, liver degeneration, yolk sac absorption delay, length decrease, and swim bladder loss, which clearly speak for acute toxicity on cardiovascular, digestive, development, and respiratory systems. The dose range of FZ-120 was lower than that used for clinical application in human beings. Moreover, UPLC-MS revealed that FZ-120 still contained toxic aconitines that were not detectable by HPLC, which might explain its acute toxicity in zebrafish. We concluded that *Fuzi* is not sufficiently safe even after long-time decoction. The zebrafish model combined with UPLC-MS assay may represent an appropriate test system to unravel aconitine-related acute toxicity.

## Introduction

As a popular complementary and alternative therapy for preventing or curing diseases, herbal traditional Chinese medicine (TCM) gains increasingly popularity around the world. Adverse effects of herbal TCM were less frequently reported than that of conventional Western medicine (Normile, 2003[21]; Drasar and Moravcova, 2004[9]; Li et al., 2004[14]). Nevertheless, appropriate use of TCM by clinical physicians and practitioners is urgently required. Otherwise side effects may occur and the erroneous use of TCM can sometimes even be serious (Abbott, 2005[1]). The quintessence of TCM is the individual prescription of herbal composition in one formula, in which the single herb interacts with each other to synergistically minimize side effects or maximize therapeutic effects. In fact, some TCM herbs exert certain toxicities, causing damages on nervous, liver, renal, respiratory system, and reproductive system. The toxic components from TCM herbs include alkaloids, anthraquinones, aristolochic acids, cardiac glycosides and others (Xu et al., 2016[33]). Among the TCM-induced side effects, aconite toxicity is of major concern, which derives from *Aconitum* species. From 1989 to 2006, over 45 aconite poisoning cases were reported in China, among which three cases were fatal (But et al., 1994[3][4]; Poon et al., 2006[24]). The cases often occur in other Asian countries, *e.g.* India and Japan (Sharma et al., 1990[26]; Ono et al., 2009[22]).

*Fuzi*, the lateral root of *Aconitum carmichaeli* Debx. (Ranunculaceae), is a distinguished TCM herb originally grown in the district of Jiangyou in the Sichuan province, China. It was first recorded in the earliest Chinese Pharmacopeia, ''*Shennongʹs Materia Medica*'' (*Shengnong Ben Cao Jing*), in the Eastern Han Dynasty of China (24−220 AD). Another Chinese medical classic, ''*Treatise on Febrile Diseases*'' (*Shang Han Lun*), elucidated its clinical effects. There are more than 20 herbal formulas containing *Fuzi* as a main ingredient, such as '*Si Ni Tang*', '*Fu Zi Tang*' and '*Zhen Wu Tang*' etc. (Chen et al., 2011[7]; Xiong et al., 2015[32]). As one of the most crucial TCM herb in the clinic, *Fuzi* has been extensively used as cardiotonic, analgesic, anti-inflammatory, and diuretic agents to treat colds, polyarthralgia, diarrhea, heart failure, beriberi, and edema for thousands of years (Murayama et al., 1991[20]; Singhuber et al., 2009[27]). However, *Fuzi*′s widespread medicinal activity is mostly accompanied with its toxicity (Chan, 2009[6]). The diester-diterpenoid alkaloids (DDAs) with acetyl group at C_8_ and benzoyl ester group at C_14_, such as aconitine, mesaconitine, and hypaconitine are the principle components responsible for the toxicity of *Fuzi*, which act as cardiotoxins and neurotoxins affecting the voltage-dependent sodium channels of cell membranes of myocardium, nerves and muscles (Chan, 2009[6]; Lin et al., 2011[16]). Water-decoction hydrolyzes DDAs into non-toxic derivatives by removing their acetyl group and benzoyl ester group (Chan et al., 1994[5]; Tong et al., 2013[29]). This is a scientific explanation, why long-time decoction has been used as a traditional way for detoxifying *Fuzi* and why it has been widely applied in many *Fuzi*-contained TCM prescriptions (Zhang, 2007[35]). 

However, in recent clinical trial, we found that the detoxified *Fuzi* still caused adverse events in patients during the treatment of osteoarthritis (Liu et al., 2016[17]). This indicates that the detoxifying effect of long-time decoction on *Fuzi* remains controversial and needs to be further ascertained. In this study, we applied both rodent and zebrafish models to thoroughly evaluate the safety of the traditional detoxification method and applied chemoprofile analyses to analyze the underlying phytochemical basis.

## Materials and Methods

### Plant materials and decoction

The clinically used decoction pieces of the lateral root of *Aconitum carmichaeli* (*Fuzi*) was obtained from Sichuan Jiangyou Zhongba Aconiti Technology Development CO., LTD (Jiangyou, China) and authenticated by the authors (voucher specimen number: JY160301). The materials were powdered and soaked in 10-fold stilled water for 30 min, followed by boiling and decoction for 60 min and 120 min, respectively. Then, the water extract was collected after filtration, and the residue was boiled and decocted with water for another 60 min and 120 min, respectively. After filtration, the second water extract was collected and mixed with the first extract, followed by concentration to dryness. The extract from 60 min decoction and 120 min decoction were labeled as FZ-60 and FZ-120, respectively. Water suspension of the powdered material of *Fuzi* was used and labeled as FZ-0 (0 min decoction).

### Chemicals and reagents

The reference standards of aconitine (Batch number: 0720-9406), mesaconitine (Batch number: 0799-9203), and hypaconitine (Batch number: 0798-9202) were purchased from the National Institute for the Control of Pharmaceutical and Biological Products (Beijing, China). The purities of the three chemicals were all above 99.8 %. Methanol, triethylamine, chloroform, and dichloromethane were of HPLC grade and commercially obtained. The distilled water was purified by Smart2Pure 6 UV/UF (Thermo Scientific, Langenselbold, Hungary).

### Mice

ICR mice with both sexes weighing 18−22 g were purchased from the Shanghai Laboratory Animal Center of Chinese Academy of Sciences (SLACCAS, Shanghai, China; Grade SPF II Certificate number: SCXK2008−0016). The mice were kept in a controlled breeding room (temperature of 22 ± 1 °C, relative humidity of 60 ± 10 %, and a 12/12 h light/dark cycle) for one week acclimatization and fed rodent laboratory chow with tap water *ad libitum* during the testing periods. All experimental procedures were in strict accordance with the China legislation on the use and care of laboratory animals.

### Zebrafish

Wide-type AB strain of Zebrafish was purchased from China Zebrafish Resource Center (CZRC), Institute of Hydrobiology, CAS (Wuhan, China) and bred by Hunter Biotechnology, Inc. (Hangzhou, China). All fishes were accredited by the Association for Assessment and Accreditation of Laboratory Animal Care (AAA LAC) International (SYXK2012-0171). After natural pair-mating and reproduction, larval zebrafish (2 dpf) were generated and housed in a light-controlled aquaculture facility with a standard 14:10 h day/night photoperiod and fed with live brine shrimp twice a day and dry flake once a day. The temperature of fish water was maintained at 28 °C (0.2 % instant ocean salt, pH6.9-7.2, conductivity 480-510 μS/cm and hardness 53.7~71.6 mg/l CaCO_3_).

### Acute toxicity assay on mice

Equal amount of male and female ICR mice were randomly divided into four groups with each 16 animals, including normal group, FZ-0 group, FZ-60 group, and FZ-120 group. All mice were fasted for 12 h with water *ad libitum* before assaying. The FZ-0 group, FZ-60 group and FZ-120 group were daily orally treated with FZ-0 (130 g/kg), FZ-60 (130 g/kg) and FZ-120 (130 g/kg), respectively, for 7 days. The normal group was daily treated with an equal volume of water for 7 days. Toxicity-related parameters were analyzed, including body weight, survival rate, weight index of main organs, histopathology of main organs, and serum biochemical (CK: creatine kinase, ALT: alanine aminotransferase, AST: aspartate aminotransferase, and LDH: lactate dehydrogenase). 

### Acute toxicity assay in Zebrafish

To determine the LC_10_ and MNLC of FZ-120, 180 larval zebrafish (2 dpf) were grouped and distributed into 6-well plates (30 fishes per well) with 3 ml water for each well. FZ-120 was mixed into the water at doses of 600, 700, 800, 900, 1000 μg/ml for oral administration for three days. Untreated well was used as normal control. During the treatment, mortality was daily recorded to generate dose-mortality response curve by using Origin 8.0 (OriginLab, Northampton, MA, USA). MNLC and LC_10_ were determined by logistic regression calculation. 

To evaluate the acute toxicity on target organs, 120 larval zebrafish (2 dpf) were orally treated with 1/10 MNLC, 1/3 MNLC, MNLC, and LC_10_ of FZ-120, respectively for 3 days. After treatment, the heart, brain, eyes, liver, intestine, spine, and behaviors of each fish were observed under the microscope. The occurrence of edema, hemorrhage, and thrombosis were also observed in the animals. All abnormal phenotypes were statistically recorded by double-blind evaluation.

### Chemoprofile analyses

HPLC analysis was performed on an Agilent 1260 Infinity HPLC system (Agilent Technologies, CA, USA). Chromatographic separation was achieved on a Hypersil BDS-C_18_ column (250 × 4.6 mm, 5 μm) (Shandon Scientific, Cheshire, UK) at 30 °C. The mobile phase consisted of methyl alcohol, water, chloroform, and triethylamine (100 : 50 : 3 : 0.15) with flow rate of 1.0 ml/min. The sample injection volume was 2 μl for FZ-0, FZ-60, and FZ-120, and the detection wavelength was 234 nm. The data was analyzed to determine the contents of aconitine, mesaconitine, and hypaconitine in all samples.

The UPLC-MS analysis was performed on an Acquity UPLC system (Waters, MA, USA) equipped with a Xevo TQS triple quadrupoleelectrospray ionization (ESI) MS (Waters, MA, USA) operated in positive ESI-mode. Chromatographic separation was carried out on an Acquity BEH C_18_ column (100 mm × 2.1 mm, particle size 1.7 μm) maintained at 40 °C.

### Statistical analysis

All tests were replicated until the experimental condition was optimized. Data were expressed as mean ± SD and subjected to one-way ANOVA, followed by Fisherʹs LSD comparison, using DPS software (Tang and Feng, 2007[28]).

## Results

### HPLC profiles of FZ decoctions

**Figure 1:**
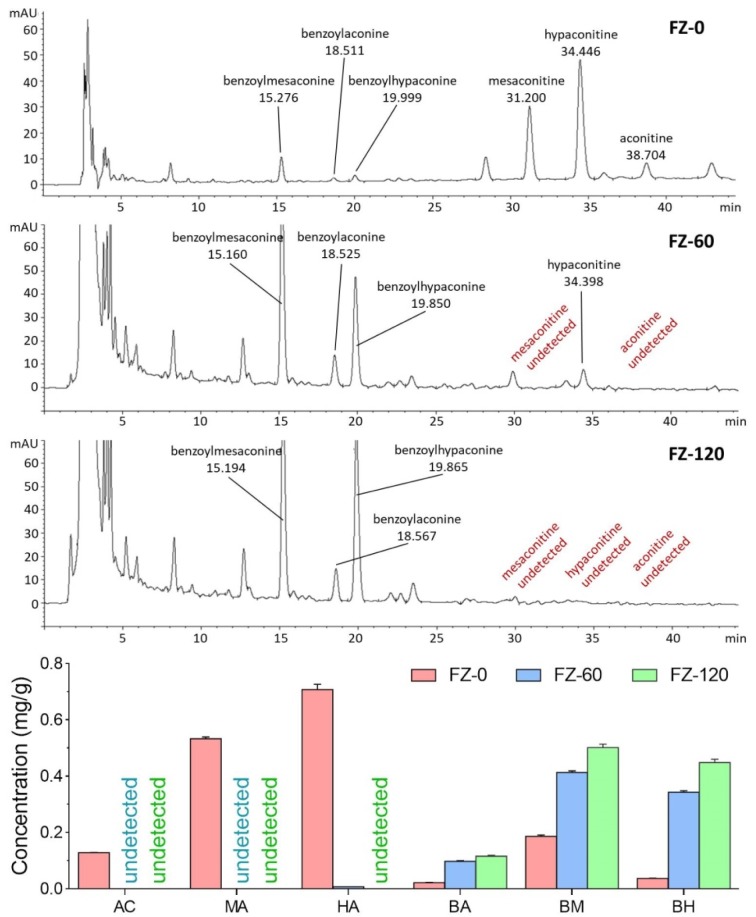
HPCL chromatograms and concentrations of aconitine components in FZ-0, FZ-60, and FZ-120

The HPLC chromatographic comparison of aconitine components among FZ-0, FZ-60, and FZ-120 is shown in Figure 1(Fig. 1). The undecocted FZ-0 sample contained the highest amounts of aconitines, such as aconitine (0.128 ± 0.001 mg/g), mesaconitine (0.533 ± 0.006 mg/g), and hypaconitine (0.708 ± 0.018 mg/g), but the lowest amounts of aconitine derivatives, such as benzoylaconine (0.022 ± 0.0002 mg/g), benzoylmesaconine (0.186 ± 0.005 mg/g), and benzoylhypaconine (0.037 ± 00007 mg/g). Upon decoction with water, the amounts of aconitines were remarkably reduced and those of aconitine derivatives increased, both in a time-dependent manner. None of aconitine and mesaconitine was found in FZ-60, and none of all the aconitines was seen in FZ-120, indicating a detoxifying effect of long-time decoction. In contrast, the highest amounts of aconitine derivatives were seen in FZ-120.

### Acute toxicity of Fuzi decoctions on mice

**Figure 2:**
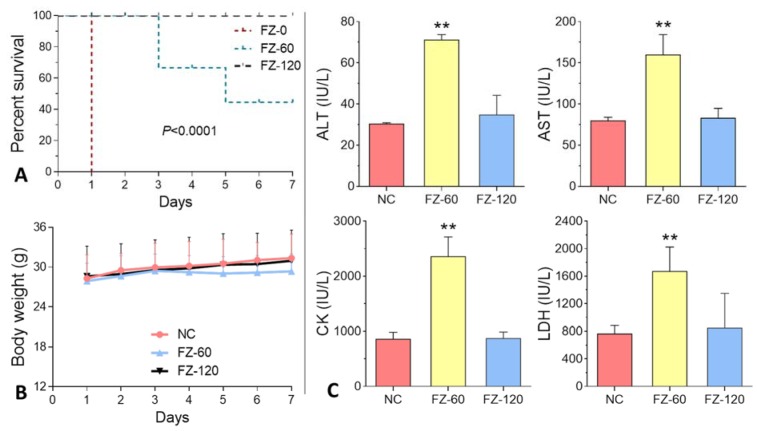
Response of mice to *Fuzi* treatments. A: survival curve; B: body weight; C: serum biochemistry (ALT: alanine aminotransferase, AST: aspartate aminotransferase, CK: creatine kinase, and LDH: lactate dehydrogenase)

**Figure 3:**
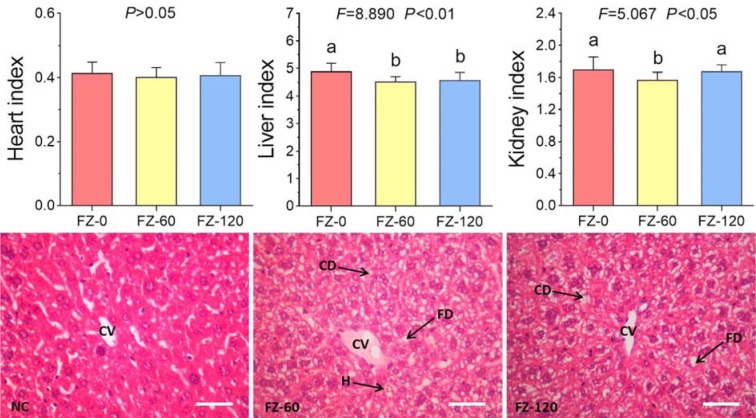
Potential toxicity of *Fuzi* in mice. Upper panel: organ indexes; lower panel: histopathological observation (CV: central vein; CD: cytoplasmic degeneration; FD: fatty degeneration; H: hemorrhage). Bar = 100 μm

As shown in Figure 2A(Fig. 2), FZ-0 induced death of all animals at day 1 after administration, and FZ-60 caused death from day 3 to 7 with a lethal rate from 33.3 to 55.6 %. In contrast, death was not observed after FZ-120 treatment. The body weight data showed that FZ-60 caused a slight decrease of body weight, while no change was visible after FZ-120 treatment (Figure 2B(Fig. 2)). Serum biochemical analyses showed that FZ-60 significantly increased the levels of ALT, AST, CK, and LDH compared to the normal controls (all *P*<0.05) (Figure 2C(Fig. 2)). However, these parameters remained normal in FZ-120-treated animals (Figure 2C(Fig. 2)). To further investigate the effect of *Fuzi* on mice, we assessed the organ indexes of heart, liver, and kidneys. As shown in Figure 3(Fig. 3) (upper panel), both FZ-60 and FZ-120 did not cause changes of the heart index, while only FZ-60 significantly decreased liver and kidney indexes (both *P* < 0.01). However, FZ-120 did not change the kidney index, but significantly decreased the liver index (*P* < 0.01). The liver abnormality induced by both FZ-60 and FZ-120 was verified through histopathological observation, in which cytoplasmic degeneration (CD) and fatty degeneration (FD) were found (Figure 3(Fig. 3) lower). Figure 2(Fig. 2) illustrates that FZ-0 and FZ-60 were toxic, but FZ-120 was safe regarding survival, body weight, and serum biochemistry of mice. Figure 3(Fig. 3) illustrates that FZ-120 possessed potential liver toxicity.

### Acute toxicity of Fuzi decoctions in Zebrafish

**Figure 4:**
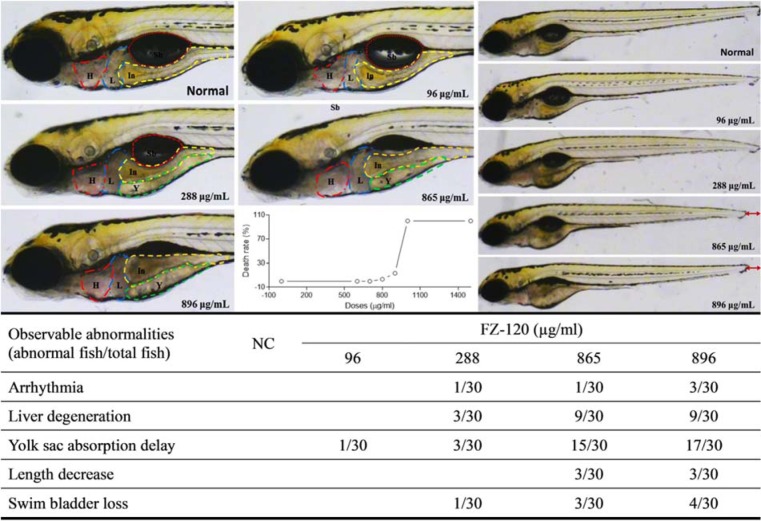
Acute toxicity observation of FZ-120 in zebrafishes. H with red line: heart; L with blue line: liver; Y with green line: yolk sac; In with yellow line: intestine; Sb with orange line: swim bladder

As shown in Figure 4(Fig. 4), FZ-120 caused the death of zebrafishes from 700 to above 1000 μg/ml, and obviously induced abnormalities of heart, liver, yolk sac, swim bladder, and body length mainly at doses ranging from 288 to 896 μg/ml. Arrhythmia (Supplementary Videos S1-S3 ) was found in one of 30 zebrafishes after treatment of 288 or 865 μg/ml FZ-120 and in three of 30 animals after treatment with 896 μg/ml FZ-120. Liver degeneration was found in 3 of 30 zebrafishes upon treatment with 288 μg/ml FZ-120 and in 9 of 30 individuals at doses of 865 and 896 μg/ml FZ-120, respectively. Yolk sac absorption was delayed in 1 of 30 zebrafishes by treatment with 96 μg/ml FZ-120, and the occurrence increased from 3 to 17 of 30 zebrafishes upon treatment with FZ-120 doses from 288 to 896 μg/ml. The swim bladder was lost in 1 to 4 out of 30 zebrafishes after treatment with FZ-120 ranging from 288 to 896 μg/ml. The body length decreased in three of 30 animals upon treatment with 865 and 896 μg/ml FZ-120. These abnormalities provide ample evidence of acute toxicity of FZ-120 to zebrafishes.

### UPLC-MS profile of FZ-120

**Figure 5:**
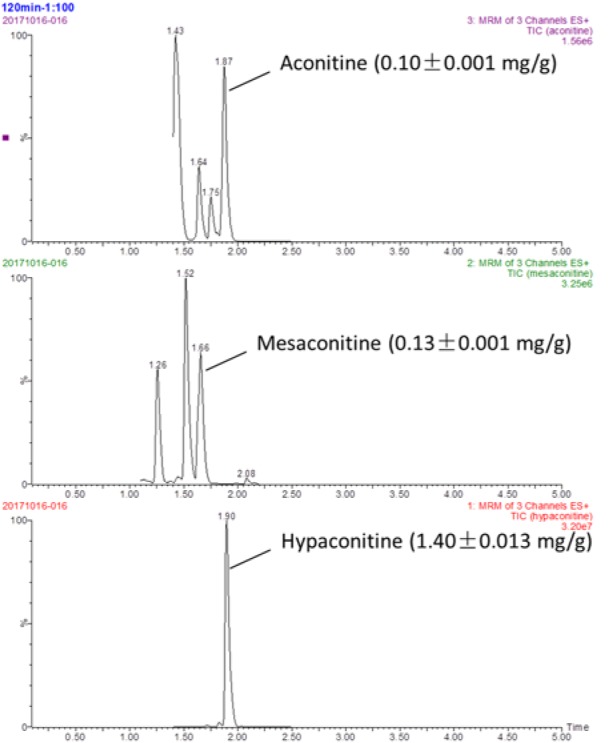
UPLC-MS analysis of FZ-120 and quantitative measurement of aconitine, mesaconitine, and hypaconitine

FZ-120 contained aconitine (0.01 ± 0.001 mg/g), mesaconitine (0.13 ± 0.001 mg/g), and hypaconitine (1.40 ± 0.013 mg/g) as determined by UPLC-MS analysis (Figure 5(Fig. 5)). These toxic components have not been detected by HPLC due to their too low contents in FZ-120.

## Discussion

Drug-induced adverse events/side effects are a major problem in clinical medicine. It also represents a major concern and remains one of the main reasons for denial of drug approval, or even withdrawal of already approved drugs from the market (William, 2003[31]). Although, a drug's toxicity has to be determined by *in vivo* mammalian screening approaches prior to approval by the authorities in accordance with the OECD guidelines (http://www.oecd.org/chemicalsafety/testing/oecdguidelinesforthetestingofchemicals.htm) and although *in vivo* animal systems exhibit high similarities with the human physiology, sometimes it remains difficult to comprehensively predict drug toxicity due to the disadvantages of low throughput, expensive and time consuming test procedures (Rovida and Harung, 2009[25]). In order to decrease the costs and time of toxicity studies, alternative *in vitro *systems have been developed using cultured cells. Despite the advantage of *in vitro* approaches that can be efficiently used for high-throughput screening, the results are still questioned due to the low accuracy of the cytotoxicity assays and the failure to recapitulate the complexity of intact organisms (Vliegenthart et al., 2014[30]). Therefore, high throughput and improved models are needed for assessing drug-induced toxicity in a variety of organ systems. Zebrafish larvae (*Danio rerio*) are such a well-established animal model, which may be well suited for toxicity assessment. It offers several advantages compared to traditional *in vivo* and *in vitro* models, including: (1) optically large and transparent body for real-time visible observation of organ response; (2) high sensitivity to toxic insults; (3) similar cellular and molecular processes as human beings; (4) high-throughput application and less experimental time due to high fecundity and rapid development; and (5) low overall costs (Hill et al., 2005[12]; McGrath and Li, 2008[18]; Vliegenthart et al., 2014[30]). Optical transparency and sensitivity to toxic agents render zebrafish larvae a very suitable organism for early prediction of drug toxicity in comparison to rodents and other larger animals. Previous studies have successfully discovered potential hepatoxicity, cardiotoxicity, and renal injury of different drugs by using zebrafish larvae (Mesens et al., 2015[19]; Liang et al., 2016[15]; Gorgulho et al., 2018[11]), demonstrating this low-order vertebrate as a suitable model for the rapid prediction of drug-induced toxicity. The overall predictive success rate of zebrafishes for drug-induced toxicity attained 100 %, being ranked as excellent (> 85 %) by the European Center for the Validation of Alternative Methods (ECVAM) criteria (Burns et al., 2005[2]; Ducharme et al., 2015[10]).

Decoction for longer time represents a traditional way to detoxify toxic TCM herbs. For example, *Fuzi* has to be pre-decocted for an extra 30 min before the conventional decoction with other herbs in one prescription (China Pharmacopeia Committee, 2015[8]). It means that *Fuzi* would be decocted for at least 1 h, but no more than 2 h before the dry out occurs. In our previous study, we evaluated the acute toxicity of long-time decocted *Fuzi* and determined its safety in mice (Tong et al., 2013[29]). Three limitations of that study are noteworthy. Firstly, the detoxified *Fuzi* was derived from an uncommonly used type (*Bai Fu Pian* without peel), but not from the commonly used type (*Hei Shun Pian* with peel). Secondly, toxic aconitines (mesaconitine and hypaconitine) still existed after long-time decoction. Thirdly, the applied body weight and death rate of mice were not adequate as parameters for the acute toxicity test. Therefore, safety of the long-time decocted *Fuzi* cannot be reliably determined, especially if it caused adverse events in the clinic (Liu et al., 2016[17]). The present study was conducted to overcome previous limitations and to determine, whether the commonly used type of *Fuzi* (*Hei Shun Pian* with peel) can be completely detoxified by long-time decoction. HPLC analysis showed that toxic aconitine components were hydrolyzed into benzoyl derivatives with increasing time of decoction, and aconitine, mesaconitine, and hypaconitine were not detected in FZ-120 (2 h decocted *Fuzi*) (Figure 1(Fig. 1)). Acute toxicity assays in mice showed that FZ-120 at 130 g/kg did not cause any deaths and did not exert side effects regarding body weight and biochemical parameters (Figure 2(Fig. 2)). These data indicate the safety of FZ-120 in mice, which are in consistent with our previous report (Tong et al., 2013[29]). However, the safety is unreal. The subsequent histopathological observations revealed an abnormal liver phenotype and a significant decrease of the liver index (Figure 3(Fig. 3)), which indicated potential hepatoxicity induced by FZ-120 in mice. A dose of 130 g/kg of *Fuzi* was safe and did not cause adverse effects in our previous report (Tong et al., 2013[29]). Apparently, this result seems to be contradictory to the results of the present study. By using a zebrafish model, we found that FZ-120 caused considerable adverse events including arrhythmia, liver degeneration, yolk sac absorption delay, length decrease, and swim bladder loss (Figure 4(Fig. 4)), indicating acute toxicity on cardiovascular (heart), digestive (liver), development (yolk sac and body length), and respiratory (swim bladder) systems. FZ-120 triggered these abnormalities ranging from 288 to 896 μg/ml. The dose range can be estimated as 28.8 to 89.6 mg/kg in human (Zhang et al., 2003[34]), which compare to a daily intake of 1.7 to 5.4 g per day for a man of 60 kg body weight. This amount is lower than the clinical dose range recommended by the China Pharmacopeia (3 to 15 g per day) (China Pharmacopeia Committee[8]). Moreover, UPLC-MS analysis revealed that FZ-120 still remained toxic aconitine components (aconitine, mesaconitine, and hypaconitine) that have never been detected by prior HPLC-based analysis (Figure 5(Fig. 5)). These components might explain the acute toxicity of FZ-120 in zebrafish. Therefore, it can be concluded that *Fuzi* at clinical doses still possesses potential toxicity to heart, liver, etc. after long-time decoction. Our conclusion covers two important points: (1) the use of *Fuzi* after long-time decoction is not sufficiently safe and urgently deserves more attention by clinicians and patients; and (2) zebrafish model combined with UPLC-MS assay may represent an appropriate test system to unravel aconitine-related acute toxicity.

Normally, patients in China obtain herbal prescriptions from clinicians and always decoct herbs by themselves. They are usually instructed to detoxify the toxic herbs by long-time decoction. Patients are enforced to take over responsibility for their own safety during the use of herbs. This approach might occasionally quite risky for the patients. Thus, sufficient knowledge about the toxicity and safety of each herb, especially the toxic ones, is urgently required by both clinicians and patients. For the first time, this study discovered the potential toxicity of *Fuzi* after long-time decoction, suggesting that the current practice of long-time decoction as only preventive measure is not adequate for aconite-related detoxification. New and more efficient approaches are needed to completely remove the toxicity of *Fuzi*. For example, co-decoction with *Radix Glycyrrhizae* (*Gancao*) can reduce the toxicity of *Fuzi* by complexation between aconitine in *Fuzi* and liquiritin in *Gancao*. Adding an excess of *Gancao* or free liquiritin makes the prescription much safer (Peter et al., 2013[23]). The combination of the co-decoction and long-time decoction offers a better strategy for *Fuzi* detoxification, warranting further investigation. Precise evaluation of toxicity is a necessary precondition prior to developing effective detoxification strategy. Owing to the insufficiency of routine methods for acute toxicity evaluation we found in this study, the toxicity of many other toxic herbs may be underestimated. Therefore, more studies are needed to apply zebrafish model and UPLC-MS assay as a new methodology to re-evaluate the safety of other toxic herbs before and after detoxification, such as *Common Monkshood* (main root of *Aconitum carmichaeli*) and *Semen Strychni* (seed of *Strychnos nuxvomica*). More toxicity we can find, more safety patients have. Further, not only the toxicity, but the efficacy of herbs should also be considered. In this study, toxic aconitine amounts were reduced with increased contents of benzoyl derivatives during decoction (Figure 1(Fig. 1)). The benzoyl derivatives of aconitines have been reported to possess therapeutic effects (Kobayashi et al., 2003[13]). Thus, further studies are needed to determine, whether or not the detoxification process alters the efficacy of *Fuzi*. *Fuzi* is one of the most typical toxic, but nevertheless useful herbs in TCM. Our study demonstrates that its current usage cannot be regarded as completely save. More efforts should be undertaken to improve the detoxification process for *Fuzi* and various other toxic TCM herbs to diminish the clinical risks for patients.

## Notes

Wan Sun, Bo Yan and Rongrong Wang contributed equally as first authors.

Jiawei Huang, Peijian Tong (The First Affiliated Hospital, Zhejiang Chinese Medical University, Hangzhou, China; tongpeijian@163.com) and Letian Shan (The First Affiliated Hospital, Zhejiang Chinese Medical University, Hangzhou, China; letian.shan@zcmu.edu.cn) contributed equally as corresponding authors.

## Acknowledgement

This study was funded by the Zhejiang Provincial Natural Science Foundation of China (Grant No: LY14H270009, LY16H270011, LY16H060005, and LY17H270016), the National Natural Science Foundation of China (Grant No: 81774331 and 81673997), the Zhejiang Provincial Major Science and Technology Project of Medical and Health of China (Grant No: 201487674), and the Zhejiang Provincial Science and Technology Project of Traditional Chinese Medicine of China (Grant No: 2013ZQ007 and 2016ZZ011).

## Supplementary Material

Supplementary material

Supplementary material Video 1

Supplementary material Video 2

Supplementary material Video 3
