# Supplementary material for: In vivo acute toxicity of detoxified Fuzi (lateral root of Aconitum carmichaeli) after a traditional detoxification process
Source: EXCLI J. 2018 Aug 31;17:889–99. doi: 10.17179/excli2018-1607 (PMC6295630; doi:10.17179/excli2018-1607)
Supplement: Supplementary material [file EXCLI-17-889-s-001.pdf]

EXCLI Journal 2018;17:889-899 – ISSN 1611-2156
Received: August 08, 2018, accepted: August 24, 2018, published: August 31, 2018

Supplementary material to:

IN VIVO ACUTE TOXICITY OF DETOXIFIED *FUZI* (LATERAL ROOT OF *ACONITUM CARMICHAELI*) AFTER A TRADITIONAL DETOXIFICATION PROCESS

Wan Sun[1,2†], Bo Yan[1,2†], Rongrong Wang[1,2†], Fucun Liu[3], Zhengyan Hu[4], Li Zhou[1], Li Yan[1,2], Kang Zhou[1], Jiawei Huang[2]*, Peijian Tong[1]*, Letian Shan[1]*, Thomas Efferth[5]

1 The First Affiliated Hospital, Zhejiang Chinese Medical University, Hangzhou, China
2 College of Pharmaceutical Science, Zhejiang Chinese Medical University, Hangzhou, China
3 Changzheng Hospital, Second Military Medical University, Shanghai, China
4 Zhejiang Provincial Center for Disease Control and Prevention, Hangzhou, China
5 Department of Pharmaceutical Biology, Institute of Pharmacy and Biochemistry, Johannes Gutenberg University, Mainz, Germany

* Corresponding authors: Jiawei Huang: hjw3657@163.com, Peijian Tong: tongpeijian@163.com, Letian Shan: letian.shan@zcmu.edu.cn

† These authors contributed equally to this work.

http://dx.doi.org/10.17179/excli2018-1607

This is an Open Access article distributed under the terms of the Creative Commons Attribution License (http://creativecommons.org/licenses/by/4.0/).

Supplementary Video 1:

Zebrafish larval in normal group with no phenotype of arrhythmia

Supplementary Video 2:

Zebrafish larval in FZ-120 group (865 µg/ml) with obvious phenotype of arrhythmia (6 s on the video)

Supplementary Video 3:

Zebrafish larval in FZ-120 group (896 µg/ml) with obvious phenotype of arrhythmia (5 s on the video)
